# Sex Differences in Liver Toxicity—Do Female and Male Human Primary Hepatocytes React Differently to Toxicants *In Vitro*?

**DOI:** 10.1371/journal.pone.0122786

**Published:** 2015-04-07

**Authors:** Milena Mennecozzi, Brigitte Landesmann, Taina Palosaari, Georgina Harris, Maurice Whelan

**Affiliations:** 1 Institute for Health and Consumer Protection, Joint Research Centre, European Commission, Ispra, Varese, Italy; 2 Center for Alternatives to Animal Testing, Johns Hopkins School of Public Health, Baltimore, Maryland, United States of America; INRA, FRANCE

## Abstract

There is increasing amount of evidence for sex variation in drug efficiency and toxicity profiles. Women are more susceptible than men to acute liver injury from xenobiotics. In general, this is attributed to sex differences at a physiological level as well as differences in pharmacokinetics and pharmacodynamics, but neither of these can give a sufficient explanation for the diverse responses to xenobiotics. Existing data are mainly based on animal models and limited data exist on *in vitro* sex differences relevant to humans. To date, male and female human hepatocytes have not yet been compared in terms of their responses to hepatotoxic drugs. We investigated whether sex-specific differences in acute hepatotoxicity can be observed *in vitro* by comparing hepatotoxic drug effects in male and female primary human hepatocytes. Significant sex-related differences were found for certain parameters and individual drugs, showing an overall higher sensitivity of female primary hepatocytes to hepatotoxicants. Moreover, our work demonstrated that high content screening is feasible with pooled primary human hepatocytes in suspension.

## Introduction

There are marked sex-based differences in the epidemiology, clinical manifestations, progression and treatment of disease, as well as pharmacodynamics, -kinetics, and adverse drug effects. These differences are derived from the fundamental biological differences between sexes and are only partially understood in molecular and cellular terms [1]. Females are under-represented in basic research as well as in animal tests, and more importantly, in human clinical trials. For many years, the Food and Drug Administration (FDA) guidelines specifically precluded participation of females in many drug studies [2] [3]. Laboratory animals are predominantly male [4], even in studies of diseases that disproportionately affect more women. Males are preferred because they are thought to be less variable due to their constant hormone levels. This variability should not be ignored as hormones can play a role in many inflammatory responses [5] [6]. Though there is considerable evidence for sex differences at all levels of biological organisation, experimental test results taken from males are assumed to be equally applicable to females [7] [8] [9] [10].

Currently, the sex of experimental animals or cells is not regularly reported in scientific publications. Overall less than 40% of studies using experimental animals and only about 25% of studies using cells in culture indicate the sex of the experimental material [11] [12].

In 2012, the American Physiological Society (APS) was one of the first bodies within the scientific publication community to announce that sex indication of the experimental material, derived from animals or humans, is required for publication in their journals [11]. This is an example of the increasing awareness on the importance of considering sex differences in drug development and preclinical studies [13]. The NIH also plans to address the issue of sex and gender inclusion across biomedical research multi-dimensionally, pointing out the need to indicate the sex of cell lines studied *in vitro* [14] and has launched a formal Request for Information (RFI) from the research community: consideration of sex as a biological variable in biomedical research.

In 2011, the European Commission established an expert group, called ‘Innovation Through Gender’, with the aim to conduct a comprehensive review on how gender analysis contributes to research. The group looked at concrete examples where appropriate treatment of gender differences enhances research and, in their report, emphasised the importance of not only studying sex differences in animal models, but also understanding sex differences in cell-based research [15].

It is important to note that "Sex" refers to the biological and physiological characteristics that define men and women, while "Gender" refers to the socially constructed roles, behaviours, activities, and attributes that a given society considers appropriate for men and women [16]. However the term gender is incorrectly becoming more commonly used in scientific publications to describe biological variation traditionally assigned to sex because scientists are not aware that a distinction exists between these terms and that this difference is an important one [17].

Clinically, women have been reported to have a 1.5–1.7 fold greater risk than men of experiencing an adverse drug reaction (ADR). Despite these reports on sex-based differences in ADR for marketed substances, the evaluation of sex differences in efficacy and toxicity has not been fully instituted for new drugs in development [18] [19]. Moreover, current *in vitro* toxicological screening of chemicals and drugs should address sex differences [20].

Specifically, acute liver failure is a rare but very serious ADR that occurs more frequently in women. In the United States, 74% of drug-induced acute liver failure occurs in women [21] [22] and the case fatality rate is about 80% [21]. Establishing more adequate drug doses on women may serve as a prevention method in the future.

The role of pharmacokinetics vs. pharmacodynamics is unclear, as is the impact of pharmacogenetics on both [23]. There are numerous theories why women experience more adverse drug reactions than men (Table 1) [24] [2] [25]. None of these theories has proven to be valid to sufficiently explain sex differences. Although gastrointestinal motility and metabolising enzymes vary by sex, studies have not consistently shown differences in drug bioavailability between men and women. The sex-based difference in plasma protein binding is postulated as a contributor to different pharmacokinetics, but the extent of this contribution has not yet been precisely defined in humans [18]. Age and number of prescribed drugs have not been confirmed as confounding factors to explain the higher incidence of ADRs among females. In these studies, females were generally similar to males with respect to age and were almost identical with respect to the number of drugs prescribed [26]. Furthermore, reporting bias does not seem to be a major factor in explaining why women experience more adverse events than men [21]. At the molecular level, many studies have reported sex differences in gene expression, protein product, or enzyme activity for cytochrome P450 and transferases without showing a clear distinct pattern [2] [27].

**Table 1:** Why women experience more adverse drug reactions than men?

Factors	Explanation as to why women experience more adverse drug reactions than men
Psychosocial and lifestyle factors [85][21]	Women take more medications than men and therefore have a higher chance of experiencing side-effects and drug-drug-interactions.
	Women report adverse events more often than men (reporting bias).
	Women are exposed in different ways to men (e.g. occupation or diet).
Differences in pharmacokinetics [86][18][62][87][88]	Dose recommendations are not sex-specific and women therefore are often overdosed.
	Bioavailability might be different due to different absorption and gastrointestinal motility.
	Tissue distribution is different because of different body composition (variations in body-weight, amount of fat, plasma volume), organ blood flow and plasma protein binding.
	Differences in metabolism (there is no firm evidence and conflicting data on varying levels of cytochrome P450 expression and activity, as well as Phase II glucuronidation rate). Despite the large variations in drug metabolism in individuals, correcting for height, weight, surface area and body composition eliminates most “sex-dependent” differences.
	Elimination rates might be different (depending on the renal excretion rates).
Differences in pharmacodynamics [18][62][86]	Differences in drug targets.
	Membrane phenomena (differences in membrane transport).
	Receptor phenomena (differences in receptor numbers and receptor binding).
	Different interactions with macromolecules.
Differences in hormones [85][89]	Influence of sex hormones, especially estrogen-effects.
	Differences in the growth hormone secretion pattern (pulsatile in male and continuous in females) from the pituitary gland with consecutive different gene-regulation.

Sex-based differences are also important for organ and cell transplants. Sex mismatch in liver transplantation increases the likelihood of graft failure, with female donor-male recipients at greatest risk [28] [29] [30] [31]. In contrast, donor sex does not affect liver transplantation outcome in children [32]. Stem cells transplants are the subject of intense studies in animal models and in clinical trials. Some animal data suggest that male and female mesenchymal stem cells may be very different [28]. Stem cell research using sex as a variable has revealed sex differences in the properties of some adult stem cells. Findings include differences in mesenchymal stem cell activation and regenerative capacity of muscle-derived stem cells [15].

The presence of an XX or XY chromosomal complement is fundamental to the genome of an individual person, animal, tissue, or cell. Every cell has a sex [33]. Therefore, based upon existing knowledge, it is inappropriate to assume that results from studies conducted on only one sex apply to the other [11]. The biological mechanisms for the greater susceptibility of women to drug-induced liver injury are still unknown.

Data on cellular sex differences are limited and are mainly based on animal studies. To our knowledge, the effect of known hepatotoxic drugs on primary human hepatocytes of both sexes have not been compared yet in a systematic manner. The objective of this study was to investigate whether sex-specific differences in acute hepatotoxicity can be observed at a cellular level by comparing the effects of well-known hepatotoxic drugs on male and female primary human hepatocytes pooled from different donor groups.

## Materials and Methods

### Chemicals and supplies

Male, premenopausal female, and postmenopausal female cryopreserved human hepatocytes, derived from 12 donors per group, were obtained from Kaly-Cell (Illkirch, France; Kaly-Cell has been authorised by the French Ministry of Higher Education and Research (Ministère de l'Enseignement supérieur et de la Recherche) to prepare and conserve human cells for scientific use according article R1243-68 of the Public Health Code. This authorisation has been renewed on 1st of September 2014; decision number AC 2014–2155) and stored in liquid nitrogen. KLC-Thawing medium and KLC-Washing medium were purchased from Kaly-Cell. Tissue culture treated, clear bottom black and white polystyrene 96 well plates were from Corning (Pero, Italy). Trypan Blue was purchased from Invitrogen (San Giuliano Milanese, Italy). Diclofenac sodium salt (CAS No. 15307-79-6), Chlorpromazine hydrochloride (CAS No. 69-09-0), Verapamil hydrochloride (CAS No. 152-11-4), Acetaminophen (CAS No. 103-90-2), Omeprazole (CAS No. 73590-58-6), and Caffeine (CAS No. 58-08-2) were purchased from Sigma-Aldrich (Milan, Italy). For cell viability, the CellTiter-Glo luminescent assay (Promega, Milan, Italy) was used. Fluorescence staining was performed with Hoechst 33342 (Invitrogen), DHE (Sigma-Aldrich), TOTO3 (Invitrogen), TMRE (Sigma-Aldrich), Fluo-4 (Invitrogen), and ER tracker Red (Invitrogen) dyes.

### Cell culture and treatment

Three groups of cryopreserved human pooled hepatocytes were used: 3F (18–45 year old female), 4F (57–74 year old females), and M (20–82 year old males). The hepatocytes were thawed following a standard procedure and counted using the Trypan blue exclusion method. The cells were seeded in 96 well plates at a density of 5000 cells/well using the Hamilton Starlet automated platform. Each group of pooled primary hepatocytes was exposed to 6 chemicals (Diclofenac, Chlorpromazine, Verapamil, Acetaminophen, Omeprazole, and Caffeine) at 8 increasing concentrations using a dilution factor 1:2 to cover a broad concentration range. The highest used concentration of each chemical corresponds to the highest soluble dose of the same chemical in culture medium. To assess the cell viability, pooled primary hepatocytes were incubated at 37°C, 5% CO2, 100% humidity for 30 min, 2h, and 5h. For hepatotoxicity endpoints, the cells were exposed to the same chemicals and concentrations for 30 min, 1h, 2h, 3h, 4h, and 5h. All chemicals were solubilised in culture medium. In each plate, 8 untreated wells per group were included. Three experimental replicates were tested. Cells treatment with drugs was completely automated and the entire process was managed by Hamilton Software (Agrate Brianza, Italy).

### Viability assay

CellTiter-Glo luminescent cell viability assay (Promega) was used to measure cell viability. Briefly, primary pooled hepatocytes were seeded in 96 well white plates and were exposed to 6 chemicals (Diclofenac, Chlorpromazine, Verapamil, Acetaminophen, Omeprazole, and Caffeine) across 8 concentrations for 30 min, 2h, and 5h. Cells were lysed with a volume of CellTiter-Glo Reagent equal to the volume of cell culture medium present in the well. After mixing the content for 2 min on a shaker, plates were incubated for 10 min at room temperature and luminescence was measured using a BMG microplate reader (BMG Labtech, Pero, Italy).

### High Content Analysis

Primary pooled hepatocytes were seeded in 96 well clear bottom black plates and were exposed to 6 chemicals (Diclofenac, Chlorpromazine, Verapamil, Acetaminophen, Omeprazole, and Caffeine) across 8 concentrations for 30 min, 1h, 2h, 3h, 4h, and 5h. In order to capture the mechanisms of toxicity, cells exposed to chemicals for 30 min and 3h were stained with dihydroethidium (DHE). Hepatocytes treated for 1h and 3h were stained with tetramethylrhodamine (TMRE) and TOTO3 dyes. 2h and 4h treated cells were stained with ER tracker red. Calcium accumulation was instead measured within cells exposed to chemicals for 5h and stained with Fluo-4. All the staining were performed in untreated and treated hepatocytes diluting each dye in growth media in presence of Hoechst 33342. After 30 min incubation with each dye at 37°C, 5% CO2 (according to supplier's instructions), and 100% humidity, live cells were imaged using the Cellomics ArrayScan VTI platform (Thermo Scientific, Pittsburgh, USA). A 10x objective was used to collect 10 image fields per well with the filter set XF93. Hoechst staining allows to quantify the number of cells and to evaluate nuclear condensation as result of cells injury. In the presence of reactive oxygen species (ROS), DHE dye is oxidised to fluorescent ethidium which intercalates into DNA; the fluorescent signal is used as a measure of oxidative stress. Mitochondrial damage was measured using TMRE dye. TMRE is positively charged and accumulates in active mitochondria due to their negative charge. Depolarised or damaged mitochondria have decreased membrane potential and fail to retain TMRE dye. Disruption of the plasma membrane was measured using TOTO-3 dye. TOTO-3 is a dead cell indicator. Only in case of plasma membrane damage it can penetrate and bind DNA. ER tracker is a cell permeant fluorescent dye, highly selective for the endoplasmic reticulum. Changes in ER fluorescence intensity can be associated to modification in the ER status. Fluo-4 was used to measure the increase in cytoplasmic calcium, a common pathophysiological event in cellular toxicity. Fluo-4 is a cell permeant calcium indicator dye. It binds to calcium and increases its fluorescent signal. All the fluorescent signals were quantified using the Target Activation Bioapplication v.4 from Cellomics Scan Software.

### Data analysis

All experiments were performed in triplicates. The data were presented as mean ± SEM. Statistical analysis was performed using GraphPad Prism version 5.1. The raw response data for cell viability and that generated by the Target Activation Bioapplication for the 6 cellular toxicity endpoints (nuclear condensation, ROS formation, mitochondrial damage, plasma membrane disruption, ER status, and calcium accumulation) were analysed using GraphPad Prism to generate dose response curves. Raw data were initially normalised to percent of maximum response and then plotted against the logarithmic of tested concentrations using nonlinear regression sigmoidal dose-response (variable slope) curve fitting. EC50 values were then calculated for each endpoint being analysed and reported in Table 2 and Table 3. In order to see if the best-fit value of LogEC50 differs between the three experimental groups (M vs 3F vs 4F), we used the F test as comparison method. This test selects the simpler model unless the extra sum-of-squares F test has a P value less than 0.05. The F test compares independent fits with a global fit that shares the same LogEC50. Moreover, statistical comparison between the fitted midpoints (LogEC50) of two curves (3F vs 4F, M vs 4F, and M vs 3F) was performed using the F test to verify whether the two curves are statistically different with respect to the LogEC50. Comparison between three groups or two groups of data were considered statistically significant when P value<0.05.

**Table 2:** EC50 values for cell viability.

	Time	EC50 (uM)			P value			
		M	3F	4F	M vs 3F vs 4F	3F vs 4F	M vs 4F	M vs 3F
**Diclofenac**	**5h**	676.70	694.70	752.40	0.2023	0.2500	0.0613	0.6715
	**2h**	671.10	757.40	702.70	0.2724	0.3689	0.4436	0.1266
	**30 min**	707.80	713	701.90	0.9846	0.8737	0.9061	0.9361
**Chlorpromazine**	**5h**	36.97	36.14	39.54	0.3004	0.1519	0.3157	0.6753
	**2h**	61.89	72.30	66.94	0.0967	0.3032	0.3082	***0.0129***
	**30 min**	100.60	111.10	80.87	***0.0430***	***0.0193***	0.1065	0.3540
**Acetaminophen**	**5h**	14.07	18.22	11.74	***0.0002***	***0.0001***	0.1058	***0.0189***
	**2h**	17.91	19.30	16.68	0.0963	0.0494	0.2587	0.2603
	**30 min**	21.59	21.51	20.51	0.8302	0.6492	0.5563	0.9700
**Verapamil**	**5h**	147.40	143.10	135.10	0.4252	0.3816	0.2272	0.6518
	**2h**	168.30	180.70	151.90	0.2705	0.1134	0.3720	0.4721
	**30 min**	214.40	150.90	157.40	***< 0.0001***	0.6113	***0.0005***	***< 0.0001***

P value< 0.05 are reported in bold and italic.

**Table 3:** EC50 values for toxicity parameters.

	Time	EC50 (uM)			P value			
		M	4F	3F	M vs 3F vs 4F	3F vs 4F	M vs 4F	M vs 3F
**Diclofenac**	**Ca 5h**	4.12	6.04	37.57	0.2105	0.6568	0.9507	0.1142
	**ER 4h**	227.70	1031	250.90	***0.0251***	***0.0134***	0.1192	0.8834
	**TOTO3 3h**	28.76	67.43	74.81	0.7071	0.9206	0.4603	0.4326
	**TMRE 3h**	839.90	542.70	716.90	***0.0061***	***0.0399***	***0.0020***	0.1755
	**ROS 3h**	431.60	83554	1762	***< 0.0001***	0.3029	***< 0.0001***	***0.0003***
	**ER 2h**	59.24	339.30	597.90	***< 0.0001***	0.4995	***< 0.0001***	***0.0030***
	**TOTO3 1h**	1056	1559	1511	0.8923	0.9946	0.9083	0.6617
	**TMRE 1h**	1430	1597	1465	0.7430	0.6169	0.4572	0.8697
	**ROS 30 min**	1182	1628	1175	***0.0004***	***0.0002***	***0.0077***	0.9579
**Chlorpromazine**	**Ca 5h**	-	-	-	-	-	-	-
	**ER 4h**	4.43	9.38	8.06	***0.0031***	0.5677	***0.0017***	***0.0346***
	**TOTO3 3h**	186.30	141	164.30	***0.0124***	0.0610	***0.0082***	0.1597
	**TMRE 3h**	36.12	22.82	56.12	***0.0395***	***0.0127***	0.2471	***0.0395***
	**ROS 3h**	-	-	-	-	-	-	-
	**ER 2h**	5.90	16.93	8.07	***0.0217***	0.0842	***0.0067***	0.4305
	**TOTO3 1h**	495.50	61.98	1333	***0.0089***	***0.0045***	0.074	0.7624
	**TMRE 1h**	90.23	79.76	237.20	0.2801	0.2322	0.8799	0.1879
	**ROS 30 min**	1.42	0.12	0.32	0.5681	0.7186	0.3023	0.5094
**Acetaminophen**	**Ca 5h**	0.76	0.65	0.77	0.9968	0.9503	0.9381	0.9859
	**ER 5h**	0.72	387.90	63.27	***< 0.0001***	0.5111	***0.0001***	***< 0.0001***
	**TOTO3 4h**	0.54	1.34	1.39	***0.0002***	0.8973	***0.0018***	***0.0020***
	**TMRE 4h**	10.80	2.12	26.92	***< 0.0001***	***< 0.0001***	***0.0001***	0.0632
	**ROS 3h**	0.71	0.70	3.44	0.8314	0.529	0.9903	0.6924
	**ER 2h**	17.59	26.92	28.16	0.3134	0.9244	0.2694	0.114
	**TOTO3 1h**	13.72	167.80	19.20	***0.0205***	0.0601	***0.005***	0.5354
	**TMRE 1h**	2.64	1.37	3.88	***0.0475***	***0.0092***	0.0824	0.3416
	**ROS 30 min**	46.67	25.15	22.36	***0.0474***	0.6031	***0.0328***	0.1283
**Verapamil**	**Ca 6h**	-	-	-	-	-	-	-
	**ER 5h**	-	-	-	-	-	-	-
	**TOTO3 5h**	617.20	425.30	681	0.2581	0.1268	0.1268	0.7627
	**TMRE 5h**	223.90	104.40	181.10	***0.0005***	***0.0013***	***0.0048***	***0.0045***
	**ROS 4h**	-	-	-	-	-	-	-
	**ER 3h**	-	-	-	-	-	-	-
	**TOTO3 2h**	1252	936.90	667.10	***0.0292***	***0.0314***	0.3175	***0.0117***
	**TMRE 2h**	555.80	611.60	557.50	0.9045	0.4088	0.7712	0.9965
	**ROS 30 min**	859.30	1518	1738	***0.0040***	0.7594		***0.0021***
**Omeprazole**	**Ca 5h**	9.41	45.57	56.77	0.2160	0.8578	0.1725	0.2187
	**ER 4h**	754.60	186.90	185.40	0.1681	0.9732	0.1397	0.0771
	**TOTO3 3h**	3855	695	539.10	0.6500	0.8649	0.5460	0.3027
	**TMRE 3h**	356.20	343.40	490.10	0.9464	0.8233	0.9789	0.7693
	**ROS 3h**	0.29	0.23	0.67	0.9279	0.8559	0.9635	0.6711
	**ER 2h**	140.70	245.50	148.90	0.1889	0.4453	***0.0445***	0.9164
	**TMRE 1h**	16.40	19.07	178.60	0.4699	0.2915	0.8590	0.2200
	**TOTO3 1h**	-	-	-	-	-	-	-
	**ROS 30 min**	-	-	-	-	-	-	-

P value< 0.05 are reported in bold and italic.

-: EC50 value could not be calculated.

## Results

### Drug selection

To test whether significant differences exist *in vitro* between human female and male hepatocytes, we selected five drugs with varying mechanisms of toxicity and documented sex-related differences in their adverse effects: Diclofenac, Chlorpromazine, Acetaminophen, Verapamil, and Omeprazole [34] [35] [22].

Diclofenac is an anti-inflammatory drug acting through cyclooxygenase (COX) inhibition. Diclofenac is metabolised in the liver by two main pathways: acyl glucuronidation (catalysed primarily by uridine 5'-diphosphoglucuronosyl transferase 2B7) and phenyl hydroxylation (catalysed by cytochrome 2C9 and 3A4). The three main metabolites are: Diclofenac glucuronide, 4’-Hydroxy Diclofenac, and 5-Hydroxy Diclofenac [36]. Diclofenac-induced hepatocyte injury is typically associated with an acute hepatitis-like histology involving necrosis, in some cases mixed hepatocellular cholestatic injury (cholestatic hepatitis), and mediated by acyl glucuronide covalent binding with cellular proteins, oxidative stress, mitochondrial permeability transition and mitochondrial damage [37] [38]. Additionally, an immuno-allergic component plays a key role in the liver toxicity of this drug [39]. There is greater susceptibility for Diclofenac related liver injury among women than men [40]. According to DrugCite, reported cases of adverse effects were 57% women and 36% men (7% unknown sex) with an average age of 57 years [41]. In 180 patients reporting Diclofenac related hepatotoxicity, 79% of cases were female and 71% of them were over 60 years of age [34]. Reported risk factors for non-steroidal anti-inflammatory drug-related hepatotoxicity in general, and especially for Diclofenac, include female sex and age above 50 years [35] [34] [42].

Acetaminophen is metabolically oxidised by cytochrome 2E1 to form a quinone imine metabolite (NAPQI), which traps cellular thiols, both protein and glutathione, by formation of covalent adducts. Acetaminophen-induced liver toxicity is characterised by apoptosis, necrosis, inflammation, and oxidative stress with the latter being the salient feature in this pathogenic process [43] [44] [45]. According to DrugCite, reported cases of adverse effects were 51% women and 32% men (17% unknown sex) [46]. A prospective study of acute liver failure at 17 tertiary care centres in the United States has shown that 73% of the patients were women and Acetaminophen overdose was the most common apparent cause of acute liver failure, accounting for 39% of all cases [22].

Chlorpromazine is a tricyclic aliphatic phenothiazine which acts by postsynaptic inhibition of dopamine receptors that formerly was the most common cause of drug-induced liver injury in the United States [47]. Chlorpromazine is activated by oxidation to electrophilic species that disrupt mitochondrial function and form adducts with cellular thiols. Approximately 10 to 12 major metabolites have been identified. The major metabolites are the monoglucuronide of N-Dedimethyl Chlorpromazine and 7-Hydroxy Chlorpromazine.

Besides being cytotoxic, Chlorpromazine induces mitochondrial injury, cholestasis, and phospholipidosis [48]. There is likely also a hypersensitivity component causing liver injury. According to DrugCite, reported cases of adverse effects in September 2013 were 64% women and 27% men (9% unknown sex), while the latest statistics (September 2014) show 43% female, 47% male, and 10% unknown sex cases [49].

Verapamil is a phenyl-alkylamine class of calcium channel blockers that may cause immune-mediated, inflammatory liver injury (hepatitis) [50]. Verapamil is subject to extensive oxidative metabolism mediated by cytochrome P450 enzymes. Furthermore, Verapamil is known to be a potent inhibitor of P-glycoprotein function. Major metabolites are Norverapamil, N-Dealkyl Norverapamil (D620), N-Dealkyl Verapamil (D617) and O-Desmethyl Verapamil (D-703) [51]. According to DrugCite, reported cases of adverse effects were 46% women and 33% men (21% unknown sex) with an average age of 58 years [52].

Omeprazole is a selective proton pump inhibitor acting by specific inhibition of the hydrogen-potassium adenosine-triphosphatase (H+, K+-ATPase) enzyme system at the secretory surface of parietal cells. Omeprazole is completely metabolised by the cytochrome P450 system to two major metabolites, 5-hydroxyomeprazole (cytochrome 2C19) and Omeprazole Sulfone (cytochrome 3A4) [53].

Adverse liver effects typically show acute centrilobular necrosis [54]. The mechanism of hepatic injury is still unknown. Statistics show that females were adversely affected in 52% and males in 45% of cases (3% unknown sex) with an age maximum for all groups of 59 years [55].

As a negative control for our study, the non-hepatotoxic compound Caffeine was selected. Caffeine is a xanthine alkaloid that is metabolised in the liver by cytochrome P450 (1A2 isozyme) into three dimethylxanthines.

### ATP measurement

**Fig 1:**
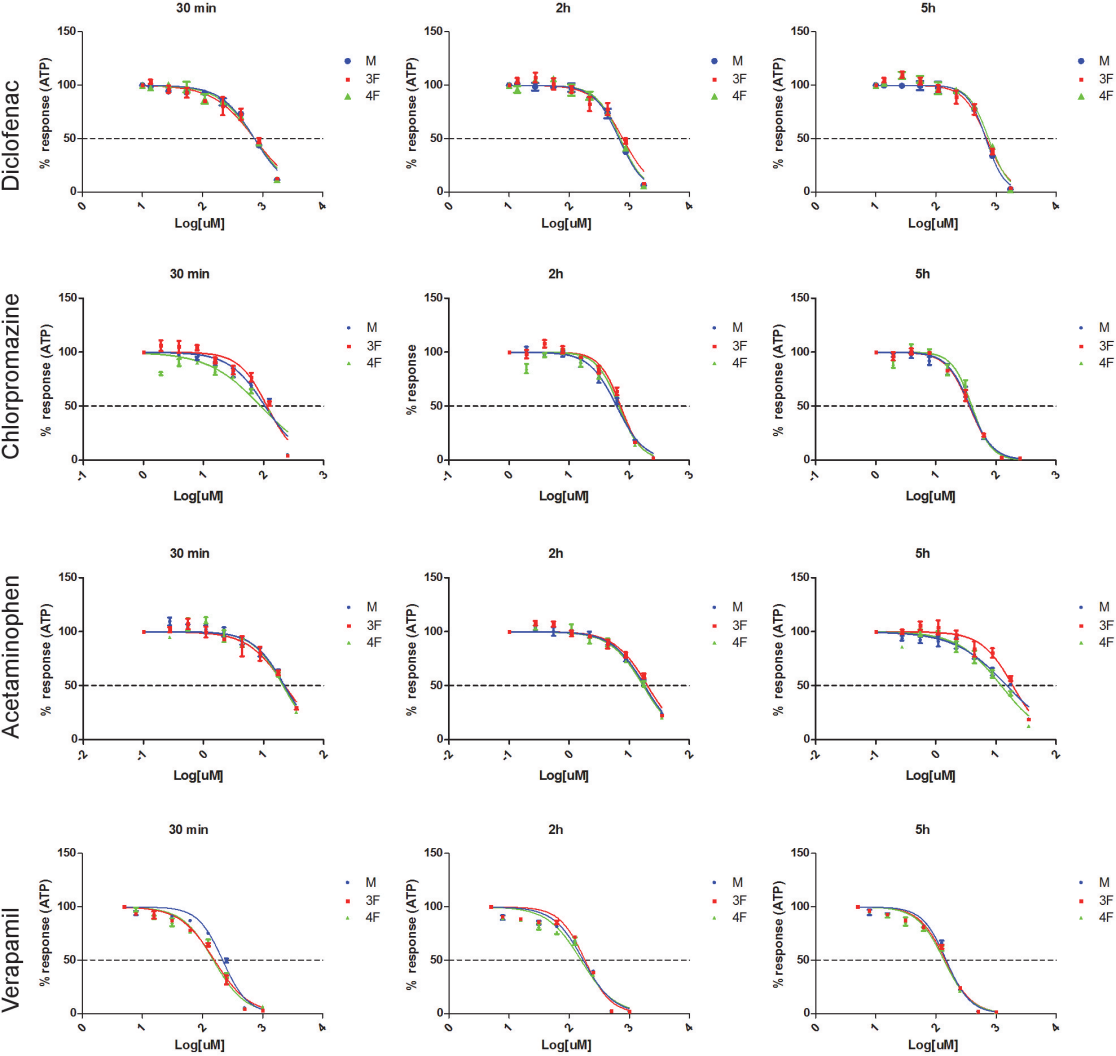
Cell viability curves for Diclofenac, Chlorpromazine, Acetaminophen, and Verapamil. 5000 human primary hepatocytes pooled from either 12 male donors (M), or 12 pre-menopausal female donors (3F), or 12 post-menopausal female donors (4F) were seeded in 96 well plates and exposed to 8 serial concentrations (using a dilution factor of 1:2) of Diclofenac (from 13.6 to 1750 uM), Chlorpromazine (from 1.9 to 250 uM), Acetaminophen (from 0.3 to 35 uM), or Verapamil (from 7.8 to 1000 uM). Decrease in cell number after 30 min, 2h, or 5h exposure to drugs was assessed by lysing the cells with CellTiter-Glo Reagent and measuring the ATP levels released. Assays were run in triplicates. Results are expressed as mean ±SEM. Dose response curves were plotted using GraphPad Prism.

Male (M), pre-menopausal female (3F), and post-menopausal female (4F) pooled primary hepatocytes derived from 12 donors per group were exposed to 8 increasing concentrations (using a dilution factor of 1:2) of Diclofenac (from 13.6 to 1750 uM), Chlorpromazine (from 1.9 to 250 uM), Acetaminophen (from 0.3 to 35 uM), Verapamil (from 7.8 to 1000 uM), Omeprazole (from 1.9 to 250 uM), and Caffeine (from 39 to 5000 uM). Cell survival after 30 min, 2h, and 5h exposure was assayed by CellTiter-Glo. Dose response curves showing a decrease in ATP levels at increasing concentration of drugs were obtained for Diclofenac, Chlorpromazine, Acetaminophen, and Verapamil at each of the tested time points (Fig. 1). EC50 values were calculated and reported in Table 2. Statistically significant differences between the three groups tested were observed only at certain time points: 30 min exposure to Chlorpromazine (P value = 0.043), 30 min treatment with Verapamil (P value<0.0001), and 5h treatment with Acetaminophen (P value = 0.0002). In particular, Verapamil (30 min) was more toxic in 4F and 3F, with no statistically significant differences between pre and post-menopausal females (3F vs 4F P value = 0.6113) (Table 2). Chlorpromazine (30 min) and Acetaminophen (5h) were instead more toxic in 4F and M compared to 3F hepatocytes. Although not statistically significant differences were observed between 4F and M for Chlorpromazine (30 min) and Acetaminophen (5h) treatment, postmenopausal cells (4F) viability measurements resulted in a lower EC50 value than that recorded for male cells: 80.87 uM (vs 100.6 uM in Chlorpromazine treated male) and 11.74 uM (vs 14.07 uM in Acetaminophen treated male) respectively (Table 2).

**Fig 2:**
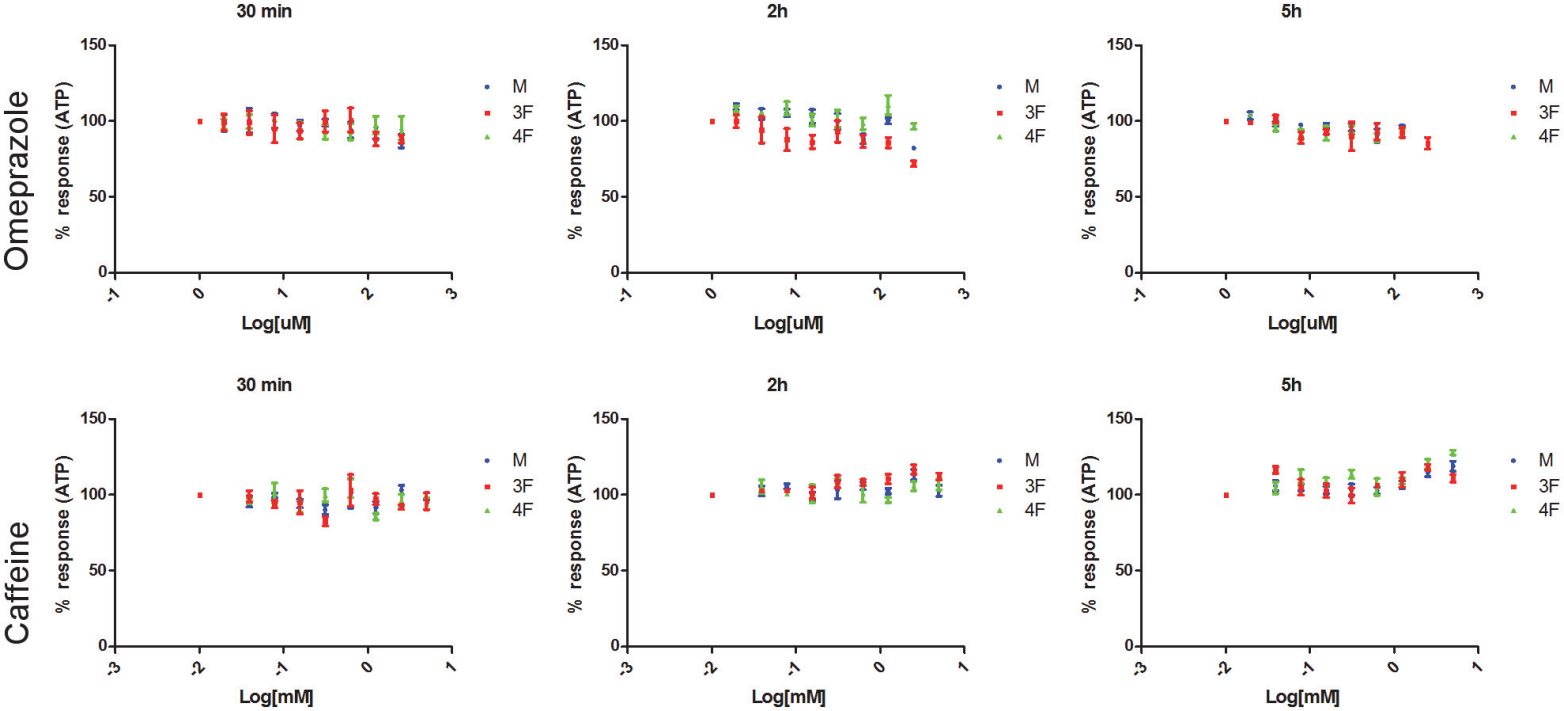
Cell viability curves for Omeprazole and Caffeine. 5000 human primary hepatocytes pooled from either 12 male donors (M), or 12 pre-menopausal female donors (3F), or 12 post-menopausal female donors (4F) were seeded in 96 well plates and exposed to 8 serial concentrations (using a dilution factor of 1:2) of Omeprazole (from 1.9 to 250 uM), or Caffeine (from 39 to 5000 uM). Cell viability after 30 min, 2h, or 5h exposure to drugs was assessed by lysing the cells with CellTiter-Glo Reagent and measuring the ATP levels released. Assays were run in triplicates. Results are expressed as mean ±SEM. Data were plotted using GraphPad Prism.

Caffeine, a non-hepatotoxic compound, and Omeprazole showed no effect on cell viability at the tested concentrations and exposure times measured (Fig. 2).

### High Content Images

**Fig 3:**
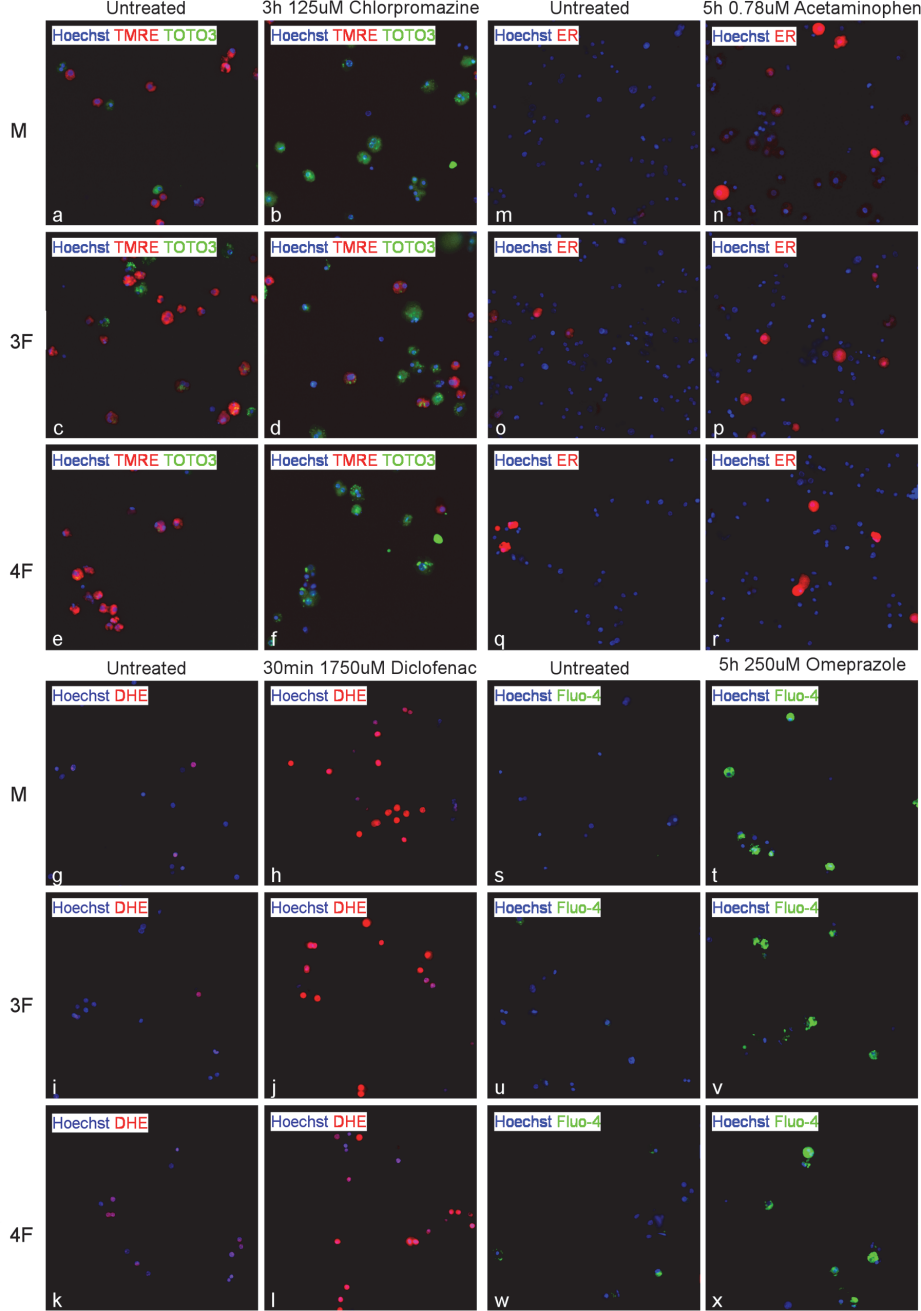
Fluorescence images of human primary hepatocytes. 5000 untreated and treated primary hepatocytes derived from M, 3F, and 4F donors were stained for 30 min using Hoechst 33342 and either TMRE and TOTO3 (a-f), or DHE (g-l), or ER tracker red (m-r), or Fluo-4 (s-x) dyes and imaged using the Cellomics ArrayScan VTI. A 10x objective was used to collect 10 images per well with the filter set XF93. Primary hepatocytes treated for 3h with 125 uM Chlorpromazine or untreated and stained with Hoechst 33342 (blue), TMRE (red), and TOTO3 (green) are reported in a-f. Primary hepatocytes treated with 1750 uM Diclofenac for 30 min or untreated and stained with Hoechst 33342 (blue) and DHE (red) are reported in g-l. Primary hepatocytes treated for 5h with 0.78 uM Acetaminophen or untreated and stained with Hoechst 33342 (blue) and ER tracker (red) are reported in m-r. Primary hepatocytes treated for 5h with 250 uM Omeprazole or untreated and stained with Hoechst 33342 (blue) and Fluo-4 (green) are reported in s-x.

We investigated the mechanisms underlying the cytotoxic effects of Diclofenac, Chlorpromazine, Acetaminophen, Verapamil, and Omeprazole by measuring changes in nuclear intensity (Hoechst 33342 dye), ROS accumulation (DHE dye), mitochondrial damage (TMRE dye), plasma membrane permeability modification (TOTO3 dye), calcium accumulation (Fluo-4 dye), and endoplasmic reticulum status (ER tracker red) in pooled primary hepatocytes. In order to capture the main mechanism of toxicity, ROS accumulation was measured at two early time points: 30 min and 3h, whereas calcium accumulation was assayed at late exposure time (5h). Mitochondrial membrane potential as marker for mitochondrial injury (at 1h and 3h) and endoplasmic reticulum status (at 2h and 4h) were measured alternating every 2 hours. Fluorescently stained live cells were imaged using the Cellomics ArrayScan VTI platform and fluorescence intensities were determined for all tested dyes using the Target Activation Bioapplication v.4 from Cellomics Scan Software. As shown in the representative fluorescence images reported in Fig. 3, hepatotoxic drugs induced formation of reactive oxygen species (increased red signal in 30 min, 1750 uM Diclofenac, Fig. 3 H-J-I compared to untreated G-I-K), induced variation of the mitochondrial membrane potential (decreased red signal in 3h, 125 uM Chlorpromazine, Fig. 3 B-D-F compared to untreated A-C-E), increased plasma membrane permeability (increased green signal in 3h, 125 uM Chlorpromazine, Fig. 3 B-D-F compared to untreated A-C-E), increased ER intensity (increased red signal in 5h, 0.78 uM Acetaminophen, Fig. 3 N-P-R compared to untreated M-O-Q), and induced accumulation of calcium (increased green signal in 5h, 250 uM Omeprazole, Fig. 3 T-V-X compared to untreated S-U-W) in male and pre/post-menopausal female. M, 3F, and 4F Caffeine treated cells were used as negative control. Caffeine treated pooled hepatocytes were stained with all the above markers and some representative images for TMRE-TOTO3 (S1 Fig., b-d-f), DHE (S1 Fig., h-j-l), ER (S1 Fig., n-p-r), and Fluo-4 (S1 Fig., t-v-x), are reported in S1 Fig.

### Nuclear intensity assessment

**Fig 4:**
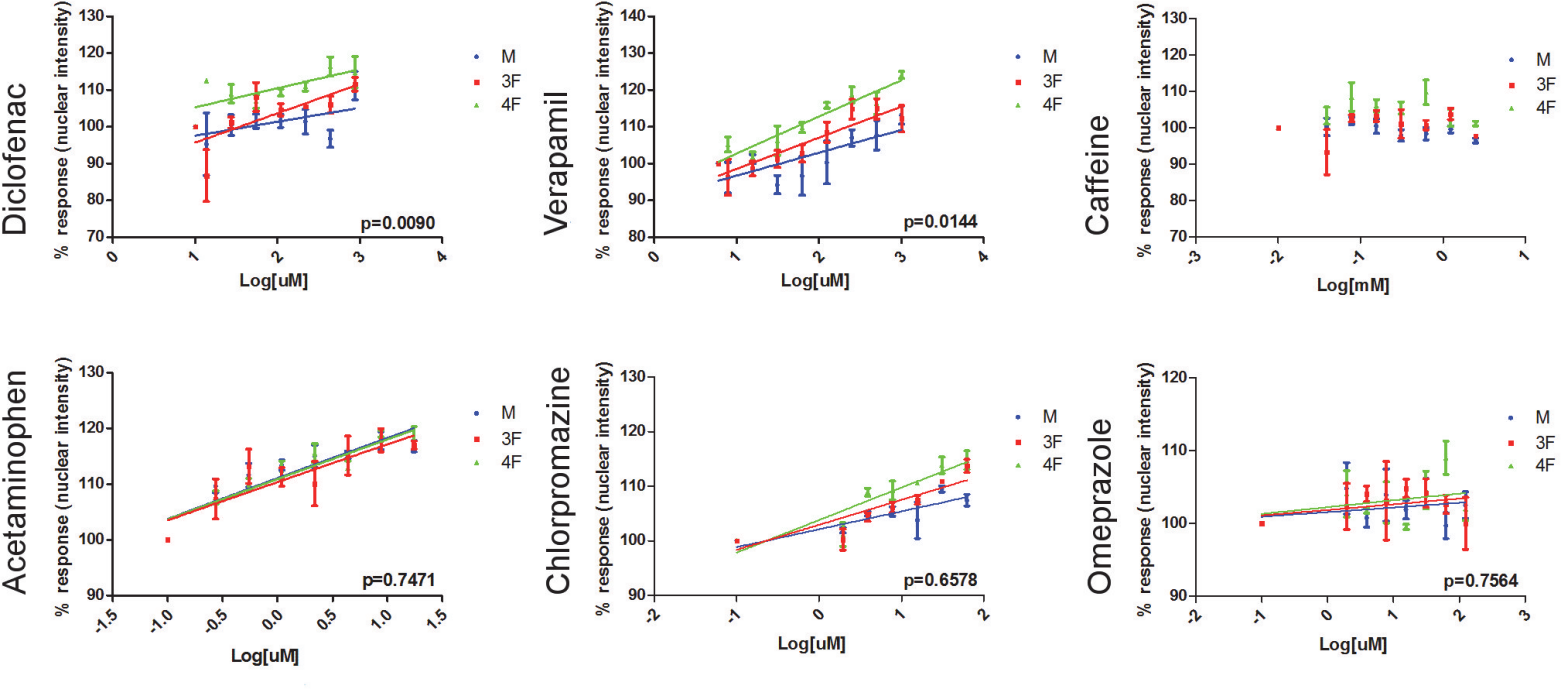
Nuclear intensity dose response curves after 4h treatment with Diclofenac, Verapamil, Caffeine, Acetaminophen, Chlorpromazine, and Omeprazole. 5000 human primary hepatocytes pooled from either 12 male donors (M), or 12 pre-menopausal female donors (3F) or 12 post-menopausal female donors (4F) were seeded in 96 well plates and exposed to 8 serial concentrations (using a dilution factor of 1:2) of Diclofenac (from 13.6 to 1750 uM), Acetaminophen (from 0.3 to 35 uM), Verapamil (from 7.8 to 1000 uM), Chlorpromazine (from 1.9 to 250 uM), Caffeine (from 39 to 5000 uM), or Omeprazole (from 1.9 to 250 uM) for 30 min, 1h, 2h, 3h, 4h, and 5h. Hepatocytes nuclei were stained with Hoechst 33342 and increase in nuclear intensity was measured at different time points using the Cellomics ArrayScan VTI platform and the Target Activation Bioapplication v.4. GraphPad Prism-derived dose response curves obtained treating primary hepatocytes for 4h with Diclofenac, Verapamil, Acetaminophen, Chlorpromazine, and Omeprazole are reported. Statistically significant sex differences (P value<0.05) comparing the EC50 values from the three groups of hepatocytes (M vs 3F vs 4F) were observed only with primary hepatocytes treated for 4h with Diclofenac (P value = 0.0090) or with Verapamil (P value = 0.0144). Diclofenac and Verapamil resulted more toxic in 4F hepatocytes. No statistically significant nuclear intensity modifications were obtained comparing M, 3F, and 4F hepatocytes treated with Acetaminophen (P value = 0.7471), Chlorpromazine (P value = 0.6578), and Omeprazole (P value = 0.7574). Caffeine did not give any increase in nuclear intensity. Assays were run in triplicates. Results are expressed as mean ±SEM.

The effect of each of the five chemicals and Caffeine on nuclear intensity was determined by staining the nuclei of primary hepatocytes with Hoechst 33342, then imaging, and quantifying the fluorescent signal. A dose dependent increase in nuclear intensity is often associated with nuclear condensation as result of cell injury. When primary hepatocytes were treated with Diclofenac, Verapamil, Acetaminophen, Chlorpromazine, and Omeprazole an increase in nuclear intensity was observed for the three groups tested (Fig. 4). Comparing the EC50 values from the three groups of cells (M vs 3F vs 4F) a P value<0.05 was obtained only after 4h treatment with Diclofenac (P value = 0.0090) and 4h with Verapamil (P value = 0.0144). At 4h, both Diclofenac and Verapamil resulted to be more toxic in post-menopausal female hepatocytes (Fig. 4). No sex differences in terms of nuclear intensity were observed at the other time points tested (30 min, 1h, 2h, 3h, and 5h). No statistically significant nuclear intensity modifications were obtained comparing M, 3F, and 4F hepatocytes at any of the tested exposure times for Acetaminophen (P value = 0.7471 at 4h), Chlorpromazine (P value = 0.6578 at 4h), Omeprazole (P value = 0.7574 at 4h), and Caffeine.

### Reactive Oxygen Species formation

**Fig 5:**
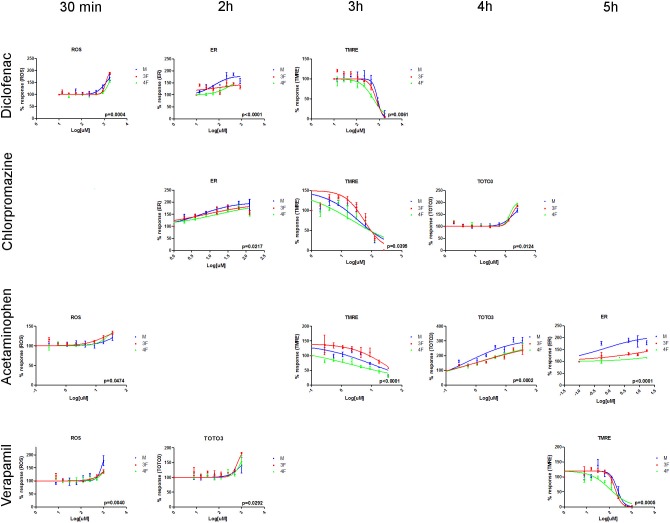
Cytotoxicity dose response curves. 5000 human primary hepatocytes pooled from either 12 male donors (M), or 12 pre-menopausal female donors (3F), or 12 post-menopausal female donors (4F) were seeded in 96 well plates and exposed to 8 serial concentrations of Diclofenac (from 13.6 to 1750 uM), Chlorpromazine (from 1.9 to 250 uM), Acetaminophen (from 0.3 to 35 uM), and Verapamil (from 7.8 to 1000 uM). ROS formation (DHE dye), endoplasmic reticulum status (ER tracker red dye), mitochondrial damage (TMRE dye), and plasma membrane permeability (TOTO3 dye) were measured at different time points (30 min, 2h, 3h, 4h, 5h) using the Cellomics ArrayScan VTI platform and the Target Activation Bioapplication v.4. Representative dose-response curves are shown for drugs having a P value<0.05 (M vs 3F vs 4F) in Table 3 (Diclofenac, Chlorpromazine, Acetaminophen, and Verapamil). Three replicates were tested and represented using GraphPad Prism. Results are expressed as mean ±SEM.

Reactive oxygen species are chemically reactive molecules containing oxygen. They are a natural by-product of the normal oxygen metabolism, but when increased and persistent they may result in significant cell damage known as oxidative stress. In primary hepatocytes treated with Acetaminophen for 30 min, we found that ROS, measured using DHE, accumulated in female cells (3F EC50 = 22.36 uM, 4F EC50 = 25.15 uM, M EC50 = 46.67 uM) at slightly significantly lower concentration than in male cells (P value = 0.0474) (Table 3 and Fig. 5). The opposite was observed in 30 min Verapamil treated hepatocytes where ROS formation occurs in male hepatocytes (3F EC50 = 1738 uM, 4F EC50 = 1518 uM, M EC50 = 859.3 uM) at significantly lower concentration than in female cells (P value = 0.0040) (Table 3 and Fig. 5). Moreover, in primary hepatocytes exposed to Diclofenac for 30 min (Fig. 3 H-J-I), we observed that ROS formation was induced at statistically higher concentration in post-menopausal female (3F EC50 = 1175 uM, 4F EC50 = 1628 uM, M EC50 = 1182 uM) compared to male and pre-menopausal female (P value = 0.0004) (Table 3 and Fig. 5). At the tested concentrations and exposure times, no statistically significant differences in ROS accumulation were observed between male and female primary hepatocytes treated with Omeprazole and Chlorpromazine (Table 3). ROS was not accumulated in primary hepatocytes exposed to Caffeine (S1 Fig.).

### Mitochondrial damage

The mitochondrial permeability and the loss of mitochondrial membrane potential with subsequent release of pro-apoptotic proteins from the inner membrane space into the cytosol, and decreased ATP production are hallmarks of cell death [56]. To investigate the involvement of mitochondria in the cytotoxic effects of Diclofenac, Chlorpromazine, Acetaminophen, Verapamil, and Omeprazole, the variation of mitochondrial membrane potential was measured in live treated primary hepatocytes using TMRE dye. As shown in Fig. 5 and Table 3, for the majority of the tested chemicals, mitochondrial damage was induced at lower concentrations in post-menopausal females compared to pre-menopausal females and males. 4F samples exposed to Diclofenac for 3h had an EC50 = 542.7 uM which is significantly lower (P value = 0.0061) than the EC50 values obtained for 3F (716.90 uM) and M (839.90 uM). Statistically significant lower EC50 values were also obtained when post-menopausal females were treated with Chlorpromazine for 3h (4F EC50 = 22.82 uM vs 3F EC50 = 56.12 uM vs M EC50 = 36.12 uM; P value = 0.0395), Acetaminophen for 4h (4F EC50 = 2.12 uM vs 3F EC50 = 26.92 uM vs M EC50 = 10.80 uM; P value<0.0001), and Verapamil for 5h (4F EC50 = 104.40 uM vs 3F EC50 = 181.1 uM vs M EC50 = 223.9 uM; P value = 0.0005) (Table 3). For Omeprazole-treated hepatocytes, no statistically significant differences between the three tested groups of cells were observed in terms of mitochondrial toxicity (Table 3). Mitochondrial membrane potential was not changed in primary hepatocytes treated with Caffeine (S1 Fig., b-d-f).

### Endoplasmic reticulum status

The endoplasmic reticulum is involved in several important functions such as the folding of secretory and membrane proteins. Various conditions including toxic effects such as exposure to free radicals, can cause pathological accumulation of unfolded proteins in the ER, a condition referred to as ER stress. ER-stress induces dilatation of ER-membranes and other alterations in ER-appearance [57] [58]. We measured ER morphological changes by staining primary human hepatocytes with ER tracker red dye and quantifying the increase in ER-signal using fluorescence microscopy imaging of live cells. Dose response curves for ER intensity showed that male hepatocytes exposed for 2h to either Diclofenac (M EC50 = 59.24 uM vs 4F EC50 = 339.3 uM vs 3F EC50 = 597.9 uM; P value<0.0001) or Chlorpromazine (M EC50 = 5.9 uM vs 4F EC50 = 16.93 uM vs 3F EC50 = 8.07 uM; P value = 0.0217), or treated for 5h with Acetaminophen (M EC50 = 0.72 uM vs 4F EC50 = 387.9 uM vs 3F EC50 = 63.27 uM; P value<0.0001) are significantly more sensitive to ER modification than pre- and post-menopausal female cells (Table 3 and Fig. 5). No statistically significant differences in terms of ER modifications were found in cells treated with Verapamil, Omeprazole, and Caffeine (Table 3 and S1 Fig., n-p-r).

### Plasma Membrane permeability

Cell membrane integrity is a well-known and common indicator of cell viability. Loss of membrane integrity was measured by quantifying the cellular influx of TOTO3 dye. In terms of plasma membrane permeability, statistically significant differences between the three groups were observed for Chlorpromazine, Acetaminophen, and Verapamil treated hepatocytes (Fig. 5). Cells treatment with Chlorpromazine for 3h induces plasma membrane damage at statistically lower concentration (4F EC50 = 141 uM vs 3F EC50 = 164.3 uM vs M EC50 = 186.3 uM; P value = 0.0124) in post-menopausal female hepatocytes confirming the data obtained by mitochondrial damage (Table 3). After 4h, Acetaminophen-treated male hepatocytes membrane disruption was induced at lower concentration (M EC50 = 0.54 uM vs 4F EC50 = 1.34 uM vs 3F EC50 = 1.39 uM; P value = 0.0002) compared to pre-and post-menopausal female cells. In accordance with the toxicity data obtained by staining hepatocytes with TMRE (5h), plasma membrane damage in female cells treated with Verapamil for 2h was induced at a lower concentration than in male samples (M EC50 = 1252 uM vs 4F EC50 = 936.9 uM vs 3F EC50 = 667.1 uM; P value = 0.0292). No statistically significant sex-differences measured as plasma membrane permeability were observed at any time point for Diclofenac, Omeprazole, and Caffeine (Table 3 and S1 Fig., b-d-f).

### Accumulation of intracellular calcium

Loss of calcium homeostasis with increased intracellular calcium level results from failure of energy dependent Ca^++—^Mg^++^ ATPase pump and it is also related to increased membrane permeability and cell mediated death. Calcium accumulation was measured in treated primary hepatocytes using Fluo-4 dye and EC50 values were obtained for Diclofenac, Chlorpromazine, Acetaminophen, and Omeprazole without finding any statistically significant differences between the three groups of cells tested (Table 3). Calcium was not accumulated in primary hepatocytes treated with Verapamil (Table 3) and Caffeine (S1 Fig., t-v-x).

## Discussion

Clinical and epidemiological data showing sex-related differences in drug-induced liver injury are well documented and, in general, a higher susceptibility in females is reported in the literature [22] [59] [21] [60] [61] [26] [62] [20] [63] [64] [42]. Basic research as well as risk assessment and clinical trials (in which women are still less enrolled and sex-specific analysis is usually not included in the results evaluation) are mainly done with males, be it from human volunteers, animals, or cell culture [7] [8] [65] [12].

This study aimed to investigate whether cellular reactions to drugs with well-known hepatotoxic effects differ between male and female human hepatocytes. Multiple parameters measured by high content imaging were used to determine sex differences in toxicity at a molecular level.

To overcome human individual genetic variability, we decided to use primary human hepatocytes pooled from 12 donors as a cellular model. Male and female pooled hepatocytes, which are solely available in suspension, were tested in cell-based assays. Female hepatocytes were further sub-classified into pre- and post-menopausal groups to account for women’s hormonal age-related changes. The age dependence of adverse effects is also reflected in clinical statistics [61] [34] [42] [26].

The three groups of human hepatocytes were exposed for a maximum of 6 hours (because of the limited life-span of hepatocytes in suspension) to five hepatotoxic drugs: Diclofenac, Chlorpromazine, Acetaminophen, Verapamil, and Omeprazole. These drugs have different mechanisms of toxicity and well documented sex-related differences in their adverse effects [34] [35] [22]. They are metabolised in the liver and, apart from Verapamil which has an idiosyncratic mechanism, their hepatotoxic effect is partly attributed to adduct formation. Hepatic injury driven by these drugs is characterised by: oxidative stress, mitochondrial dysfunction, cholestasis, phospholipidosis, and hepatitis. Moreover a non-hepatotoxic substance, Caffeine, was tested as negative control.

Previous studies of cytochrome P450 substrates for sex-dependent differences in pharmacokinetic parameters have shown inconsistent results. In our experiments, we did not check for drug metabolites to account for any differences in metabolism, although this would be an interesting addition in future studies to understand how the metabolism of these drugs contributes to differences in hepatotoxic effects.

ATP measurement showed statistically significant differences between male and females only when hepatocytes were treated for 30 min with Chlorpromazine, for 5h with Acetaminophen, and for 30 min with Verapamil. Sex differences in cell viability were not observed for the rest of the drugs and exposure times. In order to look into the mechanisms of drug toxicity, ROS formation, mitochondrial damage, endoplasmic reticulum modification, plasma membrane permeability, and calcium accumulation were measured at different time points. Dose response curves were obtained for most of the selected exposure times and, for certain parameters and specific drugs, statistically significant differences were observed between the three groups of hepatocytes tested.

Mitochondrial damage was measured *in vitro* as variation of the mitochondrial membrane potential. Our data showed that post-menopausal female hepatocytes exposed to either Diclofenac, or Acetaminophen, or Chlorpromazine, or Verapamil are more sensitive to mitochondrial damage than pre-menopausal female and male cells. These data are consistent with clinical findings showing that women are experiencing more adverse drug reactions than men and that this risk is increasing with age [26]. In women, the incidence rates of hepatic adverse drug reactions increase after menopause and this is especially valid for nonsteroidal anti-inflammatory drugs such as Diclofenac [34] [42] [35].

Moreover, progressive deterioration of mitochondrial function in postmenopausal woman has been described in various contexts [66] [67] and this could explain why most of the tested drugs showed greater toxicity in 4F cells.

*In vivo*, sex-related differences in hepatic mitochondrial function have been studied in rats and mice without showing a consistent sex-specific pattern, but rather depending on species and tissue [68] [69]. Liver mitochondria from female rats have been shown to have higher levels of reduced glutathione, higher protein content, higher cardiolipin levels, greater respiratory and phosphorylative capacities, more-energised mitochondria and to generate half the amount of peroxides than those from males [70] [68]. In mice however, no differences between males and females have been found when analysing key parameters for mitochondrial bioenergetics, oxidative stress, and apoptosis [71].

Endoplasmic reticulum status was evaluated by staining the ER and measuring its morphological changes using image quantification. For this parameter, our data showed that male hepatocytes exposed to either Diclofenac, or Acetaminophen, or Chlorpromazine are more sensitive to ER modifications compared to female hepatocytes. Sex differences in ER status were previously reported in some *in vivo* studies. For instance, it has been shown that kidneys of male mice are much more susceptible to ER stress-induced acute kidney injury than those of females [72] and that under pressure-overload condition male mice are more vulnerable to ER stress than female mice [73]. This effect has not been described in human hepatocytes before.

At first sight, our ER status data seem conflicting with the mitochondrial toxicity results, for which female cells showed higher sensitivity. A reasonable explanation could lie in the fact that ER morphology does not necessarily correlate with ER stress, it is a coping mechanism that does not always indicate stress. Induction of phospholipid biosynthetic enzymes generate new membranes, thereby increasing the volume of the ER, simultaneously diluting unfolded proteins, and preparing the compartment to receive an influx of newly synthesised folding factors [74]. Thus, increasing ER size through membrane synthesis is an integral yet distinct part of the cellular programme to overcome ER stress [75].

Disturbances in the normal functions of the ER lead to an evolutionarily well conserved cell stress response, the unfolded protein response (UPR), which is aimed initially at compensating for damage but can eventually trigger cell death if ER dysfunction is severe or prolonged ER stress involves triggering of both the “alarm” and the “adaptive” phase responses. The adaptive (protective) phase leads to the up-regulation of ER chaperone proteins which assist in the refolding of proteins, relieve ER stress, and re-establish normal ER function. The initial intent of the UPR is to adapt to the changing environment, and re-establish normal ER function [76]. Faced with persistent ER stress, adaptation starts to fail and apoptosis occurs [77]. Our data pose new questions which should be studied further to understand the differences in response to insult between sexes.

Peyrou and Cribb [78] have demonstrated that induction of ER stress proteins following a prior ER stress (‘‘ER stress preconditioning”) resulted in decreased toxicity of several model toxins and ER stress preconditioning therefore offers cytoprotection against clinically relevant nephrotoxins in renal cell lines from multiple species. Therefore we could speculate that the increase in ER intensity, observed as more pronounced in male cells, is a sign for increased protein folding, corresponded to "positive" ER preconditioning rather than to "negative" ER stress effects. Gene expression studies could provide a further insight into sex differences but were not within the scope of this study.

In correlation with the hypothesis that female hepatocytes are more sensitive to hepatotoxicant damage, we observed that in terms of nuclear condensation, Verapamil and Diclofenac are more toxic in the post-menopausal female group. Moreover for substances such as Verapamil and Chlorpromazine we showed that the plasma membrane permeability—an indicator for cell death—is more compromised in female hepatocytes than male cells. Acetaminophen treatment also showed that reactive oxygen species accumulation is occurring in female hepatocytes at lower concentration than in male cells. For cells treated with Diclofenac and Verapamil for 30 min, we instead observed that male hepatocytes have a statistically significant increase in ROS, though longer exposure to the same drugs induced mitochondrial damage mainly in female hepatocytes. Therefore, we hypothesise that male hepatocytes might be able to overcome the toxic insult induced by Diclofenac and Verapamil by increasing the intracellular level of ROS [79] [80].

For calcium accumulation no statistically significant differences between the three groups at the testing concentrations and exposure times was observed. This is probably due to the short observation period of our experiments.

To our knowledge this was the first attempt to compare *in vitro* reactions to toxicants between human male and female (pre- and post-menopausal) primary hepatocytes. Research in this field so far has been performed either *in vivo* with animal studies or *in vitro* with primary cells from animals or human immortalised cell-lines [8] [65] [7] [81] [82] [24] [27] [83] [84].

This study is the first step to elucidate cell-based sex differences in response to toxicants and the molecular pathways affected. Further experiments are needed to confirm our results and extend evidence for these observations. Some practical difficulties also have to be tackled such as the short life-span of primary hepatocytes in suspension. The time frame chosen is suitable for acute, high dose effects *in vitro*, but longer exposure times will be needed to detect more significant or long-term pathological differences between sexes. Six hours is a too short for the emergence of hepatotoxic endpoints such as steatosis. Furthermore, the differences that have been observed at early time points could become more significant after longer exposure times or in repeated dose experiments. In future experiments a broader choice of drugs might give a more comprehensive picture of differing cellular processes between male and female-derived cells. Similarly, it might be interesting to elucidate the inter-individual differences within each group by studying the responses of hepatocytes derived from single donors.

## Conclusions

In conclusion, this work presents an attempt to detect sex-based differences in cellular reactions to toxicants by using a human-relevant model *in vitro*, namely primary human hepatocytes pooled from different donor groups. Considering the fundamental difference in the genome of male and female cells it could be assumed that the cells themselves might show different sex-specific behaviour when exposed to toxic compounds, although detailed specific differences have not been demonstrated yet.

Our experiments showed significant differences in mitochondrial injury, nuclear condensation, ER status, and plasma membrane permeability between sexes presenting female cells as being more sensitive, at certain exposure times, for some of the tested drugs.

Moreover, our work demonstrated that high content screening and analysis is feasible with pooled primary human hepatocytes in suspension and such research might not only yield deeper insight into the effects of the karyotype of our basic structural and functional unit of life, but also contribute to more accurate screening methods for risk assessment that consider the varying susceptibility of male and female populations. We conclude that more focused research and data are still needed to increase evidence for male/female differences and similarities in response to toxicants, and that whenever possible, *in vitro* and *in vivo* studies should analyse data by sex to give a sex-specific report of scientific results.

## Supporting Information

S1 Fig Fluorescence images of Caffeine treated human primary hepatocytes. 5000 untreated and Caffeine treated primary hepatocytes derived from M, 3F, and 4F donors were stained for 30 min using Hoechst 33342 and either TMRE and TOTO3 (a-f), or DHE (g-l), or ER tracker red (m-r), or Fluo-4 (s-x) dyes and imaged using the Cellomics ArrayScan VTI. A 10x objective was used to collect 10 images per well with the filter set XF93. Primary hepatocytes treated for 3h with 625 uM Caffeine or untreated and stained with Hoechst 33342 (blue), TMRE (red), and TOTO3 (green) are reported in a-f. Primary hepatocytes treated with 1250 uM Caffeine for 30 min or untreated and stained with Hoechst 33342 (blue) and DHE (red) are reported in g-l. Primary hepatocytes treated for 4h with 625 uM Caffeine or untreated and stained with Hoechst 33342 (blue) and ER tracker (red) are reported in m-r. Primary hepatocytes treated for 5h with 625 uM Caffeine or untreated and stained with Hoechst 33342 (blue) and Fluo-4 (green) are reported in s-x.
(TIF)
