# Supplementary figures and images for: Sex Differences in Liver Toxicity—Do Female and Male Human Primary Hepatocytes React Differently to Toxicants In Vitro?
Source: PLoS One. 2015 Apr 7;10(4):e0122786. doi: 10.1371/journal.pone.0122786 (PMC4388670; doi:10.1371/journal.pone.0122786)

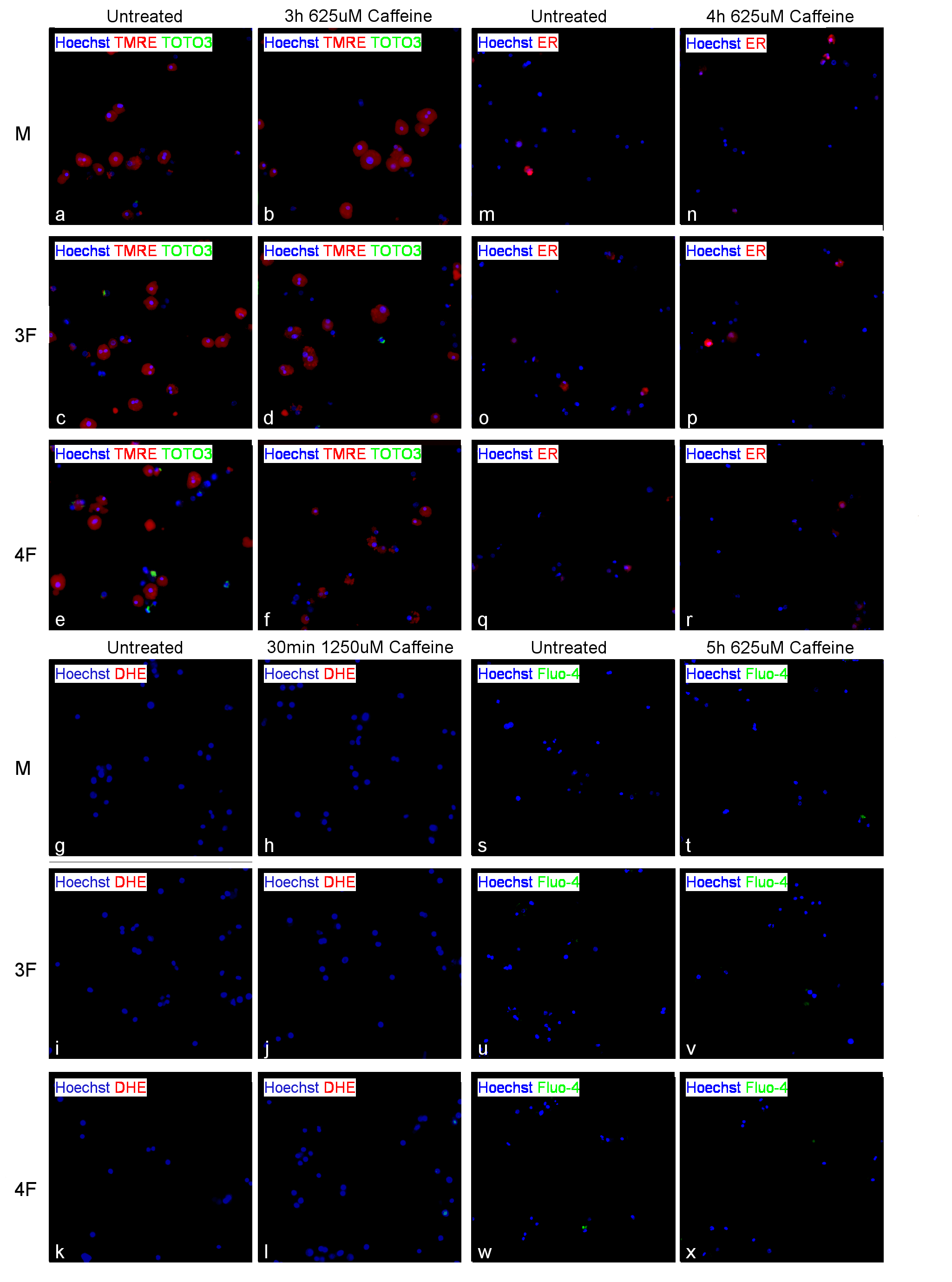

Supplement: S1 Fig — 5000 untreated and Caffeine treated primary hepatocytes derived from M, 3F, and 4F donors were stained for 30 min using Hoechst 33342 and either TMRE and TOTO3 (a-f), or DHE (g-l), or ER tracker red (m-r), or Fluo-4 (s-x) dyes and imaged using the Cellomics ArrayScan VTI. A 10x objective was used to collect 10 images per well with the filter set XF93. Primary hepatocytes treated for 3h with 625 uM Caffeine or untreated and stained with Hoechst 33342 (blue), TMRE (red), and TOTO3 (green) are reported in a-f. Primary hepatocytes treated with 1250 uM Caffeine for 30 min or untreated and stained with Hoechst 33342 (blue) and DHE (red) are reported in g-l. Primary hepatocytes treated for 4h with 625 uM Caffeine or untreated and stained with Hoechst 33342 (blue) and ER tracker (red) are reported in m-r. Primary hepatocytes treated for 5h with 625 uM Caffeine or untreated and stained with Hoechst 33342 (blue) and Fluo-4 (green) are reported in s-x. (TIF) [file pone.0122786.s001.tif]
